# Cretaceous origin of the unique prey-capture apparatus in mega-diverse genus: stem lineage of Steninae rove beetles discovered in Burmese amber

**DOI:** 10.1038/srep45904

**Published:** 2017-04-11

**Authors:** Dagmara Żyła, Shûhei Yamamoto, Karin Wolf-Schwenninger, Alexey Solodovnikov

**Affiliations:** 1 Natural History Museum of Denmark, Biosystematics Section, Zoological Museum, Universitetsparken 15, DK-2100 Copenhagen, Denmark; 2 Entomological Laboratory, Graduate School of Bioresource and Bioenvironmental Sciences, Kyushu University, Hakozaki 6-10-1, Fukuoka 812-8581, Japan; 3 Staatliches Museum für Naturkunde Stuttgart, Präparation Bernstein und Entomologie, Rosenstein 1, 70191 Stuttgart, Germany

## Abstract

*Stenus* is the largest genus of rove beetles and the second largest among animals. Its evolutionary success was associated with the adhesive labial prey-capture apparatus, a unique apomorphy of that genus. Definite *Stenus* with prey-capture apparatus are known from the Cenozoic fossils, while the age and early evolution of Steninae was hardly ever hypothesized. Our study of several Cretaceous Burmese amber inclusions revealed a stem lineage of Steninae that possibly possesses the *Stenus*-like prey-capture apparatus. Phylogenetic analysis of extinct and extant taxa of Steninae and putatively allied subfamilies of Staphylinidae with parsimony and Bayesian approaches resolved the Burmese amber lineage as a member of Steninae. It justified the description of a new extinct stenine genus *Festenus* with two new species, *F. robustus* and *F. gracilis*. The Late Cretaceous age of *Festenus* suggests an early origin of prey-capture apparatus in Steninae that, perhaps, drove the evolution towards the crown *Stenus*. Our analysis confirmed the well-established sister relationships between Steninae and Euaesthetinae and resolved Scydmaeninae as their next closest relative, the latter having no stable position in recent phylogenetic studies of rove beetles. Close affiliation of Megalopsidiinae, a subfamily often considered as a sister group to Euaesthetinae + Steninae clade, is rejected.

With more than 61 500 described species^1^, rove beetles (Coleoptera: Staphylinidae) are an outstanding example of the biological mega-diversity and the largest family of animals represented in nearly all possible terrestrial habitats worldwide^2^,^3^. Distribution of this mega-diversity among tribes and genera of Staphylinidae shows that in fact it is confined to a minority of higher taxa that are incredibly speciose^1^,^4^. Among those species-rich genera of rove beetles the genus *Stenus* that currently accounts for ca. 2 600 described species (Puthz unpublished, in Lang *et al*.^7^) is an absolute leader. And it is the second largest genus of animals as a whole, after the jewel-beetle genus *Agrilus*^8^. *Stenus* occur everywhere in all continents except Antarctica. The genus is relatively poorly represented in Australia and southern South America, and it is noteworthy by its absence in New Zealand^2,^^4^. Species of *Stenus* are obligate predators in diverse ground-based microhabitats and on vegetation, where they prey on springtails (Collembola) in particular^9^,^10^. All *Stenus* share very characteristic habitus with very large globular and protruding compound eyes correlated with active predation. The most amazing feature of *Stenus* is a prey-capture apparatus that is a harpoon-like elongated labium, which can be protruded towards a potential prey within a few milliseconds by haemolymph pressure^11^, and with the paraglossae being modified into sticky pads producing an adhesive secretion (Fig. 1a–c)^12^. Apparently this mode of predation is very efficient and unique among staphylinids and beetles in general. No one ever rigorously investigated how and why *Stenus* has evolved into one of the few biggest animal genera on Earth. But it is reasonable to assume that this unique prey-capture apparatus could be a key innovation that triggered such outstanding case of diversification.

Rigorous evolutionary explorations have not been attempted in rove beetles to a large extent because of the deficiency of phylogenetic knowledge about them. Lack of a robust rove beetle phylogeny is caused by many factors, one of which is the very limited data about their stem lineages, primarily very poor systematic understanding of the latter. For example only a few previous morphology-based phylogenetic studies on rove beetles integrated data from extinct and extant taxa^3^,^13^,^14^,^15^,^16^,^17^, and there was only a single attempt of performing a total-evidence phylogenetic analysis including fossils^16^. Such data integration is especially important since recent methodological studies (e.g. refs 18,19) indicate that a combination of phylogenetic signal preserved in recent species with palaeontological data is the most effective way to explore the history of a given organismal group, especially such an old one as Staphylinidae. Rove beetles definitely can be traced back to Middle Jurassic because the placement of the Late Triassic beetle genus *Leehermania* in the family Staphylinidae^20^ is controversial^21^.

In view of the above, it was very exciting to discover dozens of well preserved fossil specimens embedded in several pieces of Burmese amber that somewhat resembled recent *Stenus*, and had structures hinting to the presence of the *Stenus*-like prey-capture apparatus. Morphology of these amber fossils, however, did not give an easy clue about their identity. Although somewhat resembling *Stenus*, they looked substantially different and shared some characters with the subfamily Euaesthetinae that is considered a sister group of Steninae. Given that, and the mid-Cretaceous age of Burmese amber, these fossils seemed as very interesting stem lineages whose thorough phylogenetic examination could shed light on the evolution of *Stenus*, Steninae, and origin of the prey-capture apparatus. Therefore, we decided to perform a morphology-based phylogenetic study. For the correct design of such a study, the following circumstances had to be taken into consideration.

## Lacking Phylogeny of Steninae

**Figure 1:**
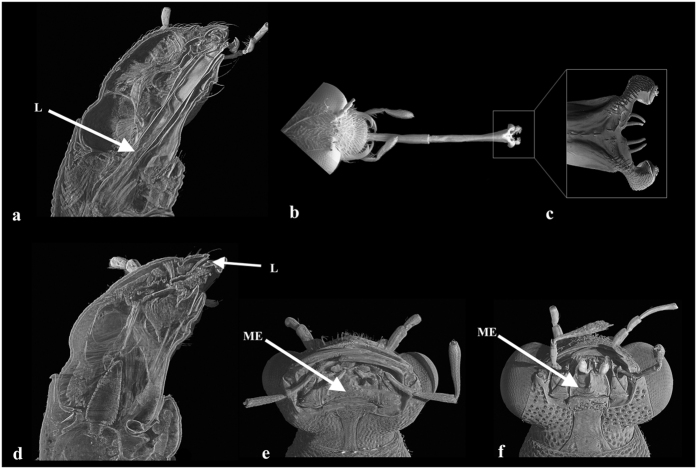
Head morphology of *Stenus* (**a**–**c**,**f**) and *Dianous* (**d**,**e**), investigated with SEM (**b**,**c**) and micro-CT (**a**,**d**,**e**), after Koerner *et al*.^24^ and Betz^57^ (modified). (**a**,**d**) Sagittal virtual section through the head. (**b**) Head with the protruded labium. (**c**) Sticky pads. (**e**,**f**) Ventral view of head. Abbreviations: L – labium, ME – mentum.

The mega-diverse genus *Stenus* is the core of the subfamily Steninae that includes two other recent genera with much poorer species diversity: the Holarctic genus *Dianous* with ca. only 220 species; and the undescribed genus with two undescribed species endemic to Australia morphologically studied in Clarke & Grebennikov^22^. *Stenus* and *Dianous* are very similar morphologically, although the latter genus often has notably smaller eyes. Also they both possess defensive glands at the tip of their abdomen that produce several compounds. Unlike other beetles with abdominal glands, the function of such glands in Steninae is not only defensive. Their pygidial gland secretion is multifunctional, allowing for an unusual way of locomotion on the water surface called ‘skimming’^23^. While the above discussed prey-capture apparatus with elongated labium is the most obvious autapomorphic feature defining *Stenus*, the labium in *Dianous* species is much shorter and does not form an adhesive prey-capture apparatus (Fig. 1d)^4^^,24^. Also *Dianous* lacks a carina on the mentum, a structure present in *Stenus* and possibly associated with the well developed prey-capture apparatus as well (Fig. 1e,f). An undescribed genus from Australia has the prey-capture apparatus and carina on the mentum too, but an overall morphology of that genus is considerably different from either *Stenus* or *Dianous*. In fact it has even smaller eyes than in some *Dianous*, and in other habitus features it rather resembles some Euaesthetinae than Steninae^22^.

At present there is not a single phylogenetic hypothesis of the origin and evolution of *Stenus*, while such data for Steninae as a whole are very limited and conflicting. The genus *Stenus* has traditionally been classified into five non-monophyletic subgenera based on several morphological characters^25^,^26^, which has recently been confirmed as an artificial division by molecular studies^24^,^27^. Currently *Stenus* is divided into a large number of presumably monophyletic species groups based on their morphology^26^ and a COI based phylogeny^24^,^27^. Similarly, the genus *Dianous* is divided into two species groups based on the morphology of the frons^28^. Members of the first group (*Dianous* group I) with large eyes similar to those of *Stenus*, have traditionally been considered as *Stenus* species. But it was recognized that they do not possess a typical *Stenus*-like prey-capture apparatus. Typical *Dianous* with small eyes are currently grouped in the *Dianous* group II^24^,^29^. *Stenus* and *Dianous* were considered as sister genera by an expert opinion^26^, and in a formal phylogenetic analysis based on the morphology^22^. The latter analysis, though, took into consideration an undescribed genus from Australia and showed *Dianous* as a sister group to the clade formed by *Stenus* and that undescribed genus. Since Clarke & Grebennikov’s^22^ target was a phylogeny of the subfamily Euaesthetinae, they indeed included Steninae as a related group. However, their taxon sampling was insufficient for rigorous phylogenetic conclusions about Steninae. In a phylogenetic study^30^ targeting sister-group relationships of the subfamily Scydmaeninae, *Dianous*, on the contrary, appears nested within *Stenus* in the analysis that used only one nuclear gene 18 S rRNA. In a molecular-based phylogenetic study, targeting Steninae, Koerner *et al*.^24^ also found *Dianous* nested within *Stenus* and therefore they suggested that the slightly protrudable labium of *Dianous* without adhesive structures is a secondary reduction of the *Stenus*-like prey-capture apparatus with sticky pads. Those authors proposed to include at least some *Dianous* species into paraphyletic *Stenus*. However, their result cannot be considered fully reliable since it was based on a single mitochondrial gene (COI). The most recent molecular-based analysis by Lang *et al*.^7^ using three genes (COI, 16 S rRNA, histone H3) confirmed Koerner *et al*.^24^ hypothesis that *Stenus* is paraphyletic with respect to *Dianous*, but with a very low posterior probability value (below 0.70). But, again, Lang *et al*.^7^ did not rigorously target the phylogeny of *Stenus* and allied taxa as such. Their aim was to test a chemotaxonomic approach presented by Schierling *et al*.^23^ that they have corroborated. All these show that a rigorous phylogenetic hypothesis for Steninae that could set a frame for the study of the origin of *Stenus* mega-diversity and testing a key innovation hypothesis for the Steninae prey-capture apparatus is still lacking.

## Controversy About Close Relatives of Steninae

Usually Steninae have been considered closely related to the subfamily Euaesthetinae in the morphology-based systematics. Sister group relationships and monophyly of these two subfamilies have been well documented in the recent phylogenetic analysis, including their respective shared morphological synapomorphies such as the unique falciform mandibles and a single pair of parasclerites in adults^22^. Also a recent molecular-based phylogenetic analysis supported a sister relationship of those two subfamilies^31^. Nonetheless, the placement of Euaesthetinae + Steninae clade (the so-called Stenine-group according to Hansen^2^) among other Staphylinine-group of subfamilies (*sensu* Lawrence and Newton^4^,^32^) still remains controversial.

Some authors^2^,^33^ considered the monotypic subfamily Megalopsidiinae as the least derived lineage sister to the Euaesthetinae + Steninae clade. But in the morphology-based phylogenetic analysis of Leschen & Newton^34^, Megalopsidiinae is placed as a sister group to the (Pseudopsinae (Steninae + Euaesthetinae)) clade. The most comprehensive morphology-based analysis by Grebennikov & Newton^30^ resolved their position even further away from the Euaesthetinae + Steninae clade, as a sister group to eight other subfamilies in the Staphylinine-group. Furthermore, in the most recent molecular phylogeny of staphylinoid beetles^31^ Megalopsidiinae was placed even further outside the Staphylinine-group of subfamilies. The mentioned morphology-based phylogeny of Grebennikov & Newton^30^ proposed Scydmaeninae as a sister group to (Steninae + Euaesthetinae), while the molecular phylogenetic studies^30^,^31^ remain insufficient in terms of gene sampling and inconclusive as far as sister-group relationships of Scydmaeninae are concerned.

## Fossil Record of Steninae and Presumably Related Subfamilies

Only two species confidently assigned to Steninae are known from Mesozoic (early Late Cretaceous), both identified as *Stenus (S. inexpectatus* Schlüter, 1978 from Bezonnais, France, and *S. imputribilus* Ryvkin, 1988 from Obeshchayushchiy Creek, Russia), described based on amber inclusion and rock impressions, respectively^35^. They are both characterized by large eyes, a three-segmented antennal club, and antennal insertions positioned on frons behind the anterior margin of eyes, a character combination matching *Stenus*. However, poor degree of preservation of these fossils makes their more confident identification and characterization impossible. So far, the majority of known fossil Steninae comes from Cenozoic deposits^36^, all of which but one, are species of *Stenus*. Among those are nine species from Baltic amber^37^ that do not differ morphologically from recent *Stenus* and share with them a typical habitus and characteristic features including the prey-capture apparatus, the latter sometimes even protruded and clearly observable. Five more Cenozoic species of *Stenus* are rock fossils described from Eocene (USA) and Oligocene (France and Germany)^38^. Even though their preservation is rather poor, they also clearly resemble recent *Stenus*^39^,^40^,^41^,^42^,^43^. The only non-*Stenus* fossil described from Cenozoic is the extinct genus *Eocenostenus* Cai, Clarke, Huang & Nel, 2014 from the late Eocene of Alès-Monteils (France). *Eocenostenus* differs from all Steninae in the strongly transverse prothorax with unusual anterolateral projections, and in the anteriorly placed antennal insertions^36^. In many other respects, however, it strongly resembles *Stenus*. Given that, a suspicion that the unusual thoracic projections and antennal position in that fossil could be an artefact caused by distortion during the fossilization of a *Stenus* specimen, should be considered.

The oldest fossil Euaesthetinae are known from the Cretaceous Lebanese and Burmese ambers^44^,^45^, and more are described from Baltic amber. Although not very abundant, these Euaesthetinae and Steninae fossils altogether provide valuable data for comprehending the origin and early diversification of this lineage.

The fossil record of Megalopsidiinae is extremely poor, and the first, and so far the only fossil representative was recently described from Burmese amber by Yamamoto & Solodovnikov^33^. The authors placed it into a recent genus *Megalopinus* and stressed the importance of that specimen for testing the sister relationships of Megalopsidiinae. Another fossil specimen from the Lower Cretaceous Lushangfen Formation in China was briefly mentioned by Cai & Huang^46^ but has not been formally described yet.

## Burmese Amber

Fossil record from the Burmese amber is of special importance since it is thought to be one of the most important sources of information about terrestrial palaeobiota in the Cretaceous^47^,^48^ even among seven major deposits of amber from that period^49^. Recently numerous rove beetle inclusions have been described from it^45^,^50^,^51^,^52^,^53^. An original habitat of the amber forest has been assumed to be a tropical Araucaria forest^54^. After some debates the age of Burmese amber is currently considered to be Late Cretaceous (earliest Cenomanian, ca. 99 Ma). So far representatives of four subfamilies of the Staphylinine-group have been described from Burmese amber: Solieriinae, Euaesthetinae, Scydmaeninae, and Megalopsidiinae.

Here, we provide the first description of the Burmese amber Steninae that is a new, extinct genus with two new species. To solve the systematic position of these Burmese amber specimens we conducted a phylogenetic analysis that included several fossil and recent taxa analysed together. Primarily we used the morphological dataset of Clarke & Grebennikov^22^. Even though this dataset was mostly developed for testing the monophyly of Euaesthetinae and resolving their internal relationships without broad sampling of Steninae, its character list is so far the most complete and appropriate for our study. We also aimed to test the monophyly of the Euaesthetinae + Steninae clade while adding a potential stem group fossil to the analysis. Because of Hansen’s^2^ hypothesis, and an overall controversy about Megalopsidiinae sister-group, we added to our analysis a recent species from this subfamily and its only fossil *Megalopinus extinctus* Yamamoto & Solodovnikov, 2016. Simultaneously we tested the phylogenetic position of *M. extinctus* hypothesized in its original descriptive paper^33^.

## Results

**Figure 2:**
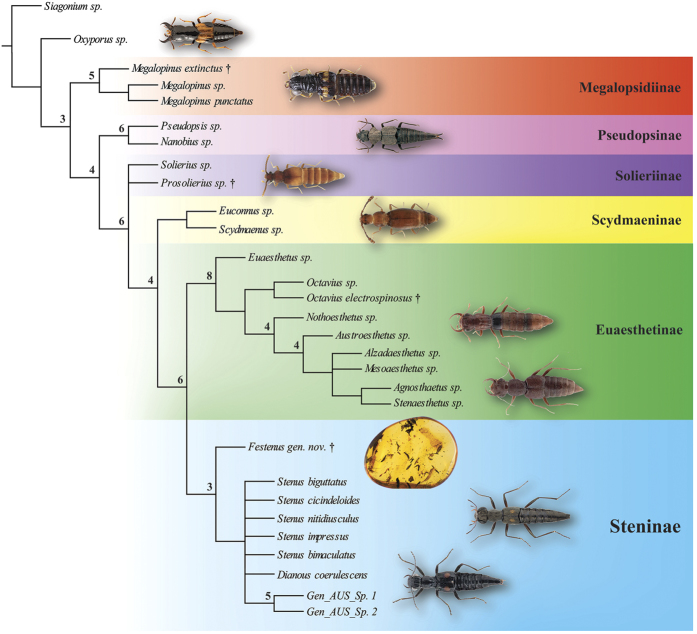
Phylogenetic position of *Festenus* gen. nov.: strict consensus of the 15 most parsimonious trees. Numbers at nodes represent Bremer supports. Picture of *Oxyporus maxillosus* (Fabricius) by M.E. Smirnov (www.zin.ru/Animalia/Coleoptera), *Stenus biguttatus* by K.V. Makarov (www.zin.ru/Animalia/Coleoptera), *Megalopinus* sp. by Yamamoto & Solodovnikov^33^. Pictures of *Zalobius spinicollis* LeConte (Pseudopsinae), *Solierius obscurus* (Solier, 1849) (Solieriinae), and *Veraphis* sp. (Scydmaeninae) from Grebennikov & Newton^30^ (modified, https://creativecommons.org/licenses/by/4.0/). Pictures of *Agnosthaetus* sp., *Stenaesthetus* sp. (both Euaesthetinae), and *Dianous nitidulus* LeConte (Steninae) from Clarke & Grebennikov^22^ (modified).

**Figure 3:**
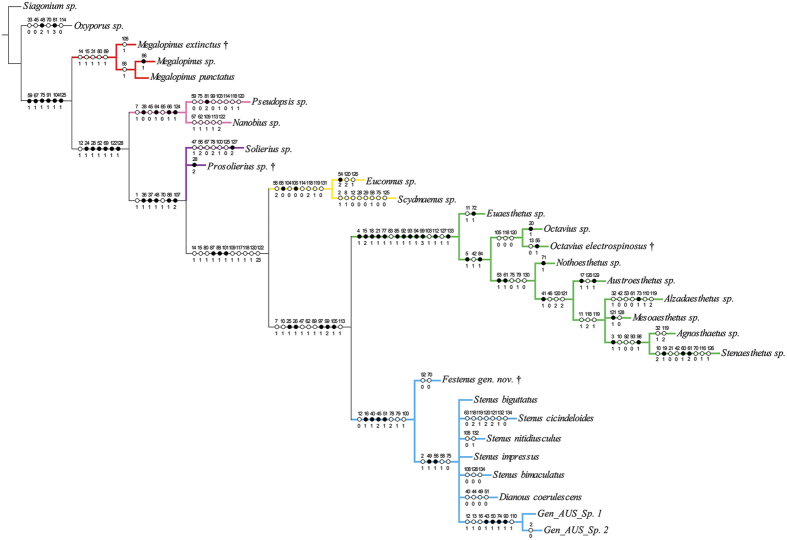
Character support for phylogenetic position of *Festenus* gen. nov.: strict consensus of the 15 most parsimonious trees. Circles with numbers along branches indicate unambiguously optimized synapomorphies (autapomorphies for terminal branches): black, unique changes; white, homoplasious changes; character numbers above circles, character state numbers below circles.

**Figure 4:**
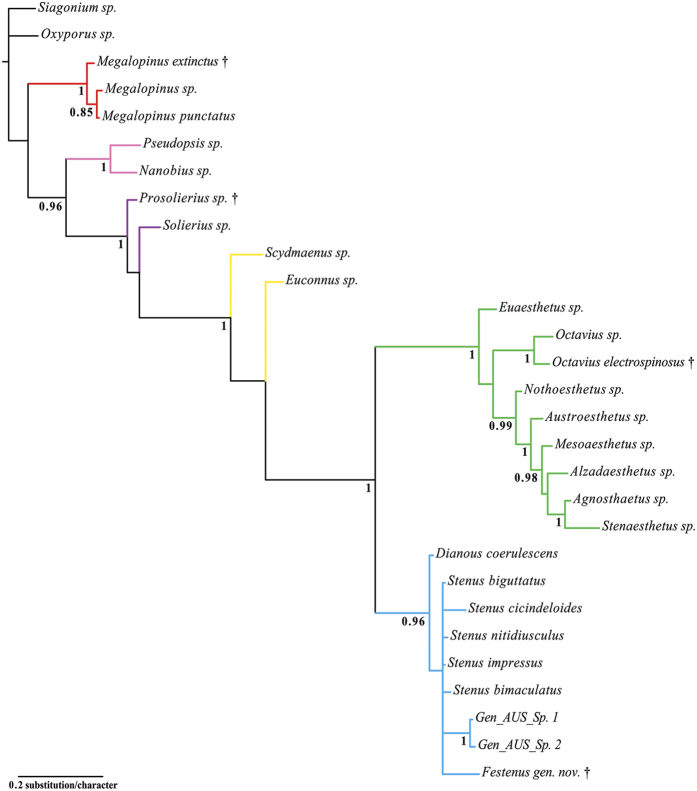
Fifty percent consensus tree from a Bayesian analysis with gamma rates. Posterior probabilities greater than 0.84 reported below the corresponding node.

The maximum parsimony analysis under equal weights (EW) resulted in 15 most parsimonious trees (MPTs) with 304 steps, consistency index (CI) = 0.50, and retention index (RI) = 0.80. A strict consensus tree is shown in Figs 2 and 3. Re-analysis under implied weights (IW) and at each *k*-value resulted in a single most parsimonious tree in each run (the tree obtained at *k* = 3 is shown in the Supplementary Fig. S1). The BI analysis (Fig. 4) converged before 15 million generations, and at the end of the run an average standard deviation of split frequencies had stabilized well below 0.01, while nearly all PSRF values were 1.000 (maximum 1.002).

All types of analyses and methods consistently revealed the strongly supported (Pseudopsinae + (Solieriinae + (Scydmaeninae + (Euaesthetinae + Steninae)))) clade, but differed in the position of Megalopsidiinae. MP analysis showed Megalopsidiinae as a sister to the above-mentioned consistent clade (Bremer support = 3), while Oxyporinae as a sister group to that including Megalopsidiinae (Fig. 2). However, BI analysis showed no support for that (gamma rates, Fig. 4), or placed Megalopsidiinae in an unsupported clade with Oxyporinae (equal rates, Supplementary Fig. S2). In our analysis, monophyly of Euaesthetinae and Steninae, as well as their sister relationships were recovered with strong support (Bremer support = 6, PP = 1.00, and 5 synapomorphies −25-1, 26-1, 97-1, 99-2, 105-1). All analyses revealed Scydmaeninae as a sister group to the Euaesthetinae + Steninae clade (Bremer support = 4, PP = 1.00, and 3 synapomorphies −87-1, 88-1, 101-1). The monogeneric subfamily Solieriinae formed a sister group to the (Scydmaeninae + (Euaesthetinae + Steninae)) clade (Bremer support = 6, PP = 1.00, and 5 synapomorphies −36-1, 37-1, 48-1, 86-1, 107-2), while Pseudopsinae was shown as a sister group to all of them (Bremer support = 4, PP = 0.96, and 6 synapomorphies −24-1, 28-1, 52-1, 69-1, 122-1, 128-1). Differences among analyses (between EW and IW with k = 3, as well as between BI with rates set to equal and gamma) were related to the resolution of the Steninae clade. While EW parsimony and BI with equal rates showed our extinct genus as a stem to both recent genera, the IW parsimony (k = 3) and BI with gamma resolved *Dianous* as a sister group to *Stenus* that included the fossil genus. Nevertheless, in every analysis, the new extinct genus was recovered as a representative of the subfamily Steninae, even though its morphology combines characters of Euaesthetinae and Steninae.

An overall topology of all trees resulted from the MP and BI analyses here is similar to that from Grebennikov & Newton^30^, even though the latter authors used more diverse set of characters (89 of larvae and 122 of adults), and their character list and taxon sampling differed from ours. Also our test of the impact of the autapomorphic characters in Bayesian inference revealed the same topology with and without autapomorphies. However, some nodes were slightly better supported when autapomorphies were included into analysis.

**Systematic palaeontology**

Order Coleoptera Linnaeus, 1758

Family Staphylinidae Latreille, 1802

Subfamily Steninae MacLeay, 1825

Genus *Festenus* gen. nov. LSID, urn:lsid:zoobank.org:act:EF495C63-E977-4FB4-88B8-DD3C177AB446

Type species *Festenus robustus* sp. nov.; by present designation.

Etymology: The new genus name is a chimera formed from the Latin word *festum* (meaning feast, carnival, holiday), and the genus name *Stenus*. It reflects the fact that the new genus resembles *Stenus* and apparently liked to gather, as shown by the amazing amber specimen from Stuttgart Museum that captured 15 beetles.

Diagnosis: Distinguishable from all extant species of Steninae by the following combination of character states: antennal insertion near frontal margin of head, anteriorly to eye (also present only in the undescribed extant Australian genus); submentum and gula separated by suture located significantly anterior to posterior tentorial pits; pronotal marginal carina reaching anterolateral prothoracic margin; pronotosternal suture present; intermesocoxal process of mesoventrite overlapping intermesocoxal process of metaventrite ventrally; elytra with epipleural keel.

**Description:**

Small (body ca. 3 mm long); body elongate, slender, moderately flattened.

Head: Head slightly elongate, with distinct lateral and dorsal neck constriction; antennal insertion exposed and positioned anterior to eyes. Antennae filiform, 11-segmented, setose; antennomere a11 acuminate, ca. 1.5 times as long as a10, antennal club present, 3-segmented; a1 and 2 distinctly thicker than subsequent few antennomeres; a3 distinctly longer than a2 or a4, each. Labrum large, with anterior margin smooth and straight. Mandibles slender, falciform, tips concealed beneath labrum when mandibles closed; mandibular molar lobe absent. Maxillary palpi 4-segmented; all palpomeres (p) elongate; p1 slender and shorter than p2; p3 subequal in length to p2, wider and densely setose; p4 minute, hyaline, setose. Labial palpi half as long as maxillary palpi, 3-segmented all segments elongate; p2 longer than either p1 or p3, strongly expanded, subglobular; p3 acicular, hyaline; labial insertion almost contiguous. Adhesive cushions of labium present. Mentum with medial longitudinal carina. Submentum and gula separated by suture. Gula well developed with sutures distinctly separated from each other.

Thorax: Pronotum quadrangular or rectangular; with complete pronotal marginal carina and complete pronotosternal suture. Mesoventrite with midlongitudinal carina. Mesothoracic anapleural suture present. Intermesocoxal process of mesoventrite overlapping intermesocoxal process of metaventrite ventrally. Mesal posterior lobes of metaventrite present. Scutellum with at most a minute portion exposed. Elytra slightly elongate, or almost as long as wide; without striae, but with epipleural keel present. Hind wings not visible. Procoxae subconical, narrowly separated, with carina-delimited groove on procoxal mesial surface. Protibia rounded in cross section, without external spines. Metacoxae subconical, not strongly expanded laterally, half as long as wide, widely separated from each other. Tarsal formula 5-5-5; tarsi elongate, tarsomeres 1–3, slightly, tarsomere 4 more strongly lobed anteriorly, gradually decreasing in length. First tarsomere of posterior tarsi longer than apical tarsomere. Tibial apical spurs reduced. Tarsal claws smooth, simple, slender, and slightly curved. Empodial setae and ventral process projecting over empodium absent.

Abdomen:

Abdominal intersegmental membrane attached preapically to preceding segment, with hexagonal membrane sclerites. Segments III-VI with one pair of paratergites; segment VII without paratergites. Apex of sternite IX in male not acutely produced.

*Festenus robustus* sp. nov. LSID, urn:lsid:zoobank.org:act:FF5FAD2E-CAE4-4D26-9532-4BA2CD7BDC85

Figures 5 and 6.

Material: Holotype. Yamamoto collection, AMNH Bu-SY10/19. Paratype(s): Yamamoto collection, AMNH Bu-SY10/18, AMNH Bu-SY11/25.

Etymology:

The name refers to a slightly more robust habitus of this species compared to its congener *F. gracilis*.

Locality and horizon:

Myanmar, Kachin, Hukawng Valley, Burmese amber; Upper Cretaceous, lowermost Cenomanian^49^.

Diagnosis:

Habitus robust. Pronotum longer than wide (rectangular), smooth. Eye surface botryoidal; eyes small, not reaching posterior margin of head. Abdomen wide; tip of abdomen rounded.

Description:

Body wide, ca. 3 mm in length. Head, pronotum and elytra coarsely sculptured. Head slightly narrower than elytra, 0.4 mm in length (from apex of labrum to anterior margin of pronotum), 0.6 mm in width (including eyes) [both head measurements taken from specimen AMNH Bu-SY11/25]. Neck wide and short. Antennae reaching anterior margin of elytra, club antennomeres 9–11, each, longer than wide, narrower posteriorly and gradually wider towards apex, length of a9–0.1 mm, a10–0.09 mm, and a11–0.07 mm [taken from holotype, AMNH Bu-SY10/19]. Pronotum almost quadrangular, 0.5 mm in length, and 0.6 mm in width [taken from specimen AMNH Bu-SY11/25], widest at middle, gradually narrowing anteriad and posteriad, with 2 longitudinal carinae (furrows). Elytra 0.7 mm in length and 0.9 mm in width (from posterior margin of pronotum to anterior margin of elytron) [taken from specimen AMNH Bu-SY11/25], anterior margins rounded. Legs more robust than *F. gracilis*, length of protibia −0.4 mm, mesotibia −0.5 mm, and metatibia −0.6 mm; length of mesotarsus −0.3 mm, metatarsus −0.4 mm [all leg measurements taken from holotype AMNH Bu-SY10/19]. First tarsomere of posterior tarsi longer than apical tarsomere. Abdomen wide. Tergites III-VII with wide, very distinct paratergites. Tergite III with a row of inverted ‘U-shaped’ carinae basally.

*Festenus gracilis* sp. nov. LSID, urn:lsid:zoobank.org:act:0A167072-A6BD-45F7-BC6F-0790A3471785

Figure 7.

Material:

Holotype: SMNS Bu-119/1. Paratypes: SMNS Bu-119, specimen no 4, 7, 9, 10, 12,13, 14, 15.

Etymology:

The name refers to a slightly more gracile habitus of this species compared to its congener *F. robustus*.

Locality and horizon:

Myanmar, Kachin, Hukawng Valley, Burmese amber; Upper Cretaceous, lowermost Cenomanian^49^.

Diagnosis:

Body slender. Pronotum almost quadrangular, with carinae. Eye surface smooth; eyes occupying more than half of lateral head margin, reaching posterior margin of head. Abdomen slender, with pointed tip.

Description:

Body narrowly elongate, 3.3 mm in length [all measurements taken from holotype, SMNS Bu-119/1]. Black, with posterior half of elytra brighter (brown). Head narrower than elytra, 0.4 mm in length (from apex of labrum to anterior margin of pronotum). Frons slightly concave, longitudinal elevations (furrows) present. Eyes large, flat, reaching posterior margin of head. Antennae reaching posterior margin of pronotum, club segments slightly longer than wide, each segment narrower posteriorly and gradually wider towards apex; length of antennomere 9–0.05 mm, antennomere 10–0.05 mm, and antennomere 11–0.08 mm. Gular sutures separated. Pronotum longer than wide, 0.5 mm in length, with 2–3 longitudinal carinae (furrows) (might be a result of squishing). Elytra 0.8 mm in length, with rounded humeri, seemingly shallow humeral impressions reaching half of elytra length. Legs slender, length of pro- and mesotibia −0.4 mm, pro- and mesotarsus −0.25 mm. Tarsomere 4 of all tarsi seem slightly bilobed. Abdomen gradually tapered towards apex.

## Discussion

### Placement of the new genus within Euaesthetinae + Steninae clade

The new genus shares with Steninae + Euaesthetinae several morphological features that were pointed out as synapomorphies of that clade by Clarke & Grebennikov^22^: slender, falciform mandibles with their tips concealed beneath labrum when mandibles closed; maxillary palpomere 3 densely setose and without macrosetae; maxillary palpomere 4 minute, hyaline; labial palpomere 2 strongly expanded, subglobular or subfusiform; labial palpomere 3 acicular, hyaline; absence of empodial setae; and preapical attachment of abdominal intersegmental membrane to preceding segment. At the same time it differs from Steninae and most of the genera of Euaesthetinae in two characters: presence of elytral epipleural keel; and absence of ventral process projecting over empodium. Presumably these features of our stem lineage are plesiomorphies that were lost in the course of the Steninae-Euaesthetinae evolution. The new genus also has several characters not found in recent Steninae, but at the same time occurring in Euaesthetinae and not listed as synapomorphies of the latter subfamily according to Clarke & Grebennikov^22^: pronotal marginal carina not meeting pronotosternal suture, reaching anterolateral prothoracic margin; presence of pronotosternal suture; and intermesocoxal process of mesoventrite overlapping with intermesocoxal process of metaventrite ventrally. The new genus possesses one character that is synapomorphic for the subfamily Steninae: labial palps inserted close together and near anterior margin of labium; and two others that are synapomorphic for the genus *Stenus*: presence of adhesive cushions of labium; and mentum divided by medial longitudinal carina, in both cases synapomorphies are according to Clarke & Grebennikov^22^. One character, which is traditionally used as diagnostic for the subfamily Steninae, namely location of the antennal insertion on frons between eyes, has a different state in the new genus that has antenna inserted near the frontal margin of head, anterior to eye. However, this state is also known within Steninae in the undescribed recent Australian genus, where it was interpreted as a reversion^22^, and in the extinct genus *Eocenostenus*. The most remarkable feature of the new genus is presence of characters that are associated with the prey-capture apparatus: sticky pads and carina on the mentum, so far known only in *Stenus* and the undescribed Australian genus.

### Closest relatives of Steninae

The subfamilies Steninae and Euaesthetinae are morphologically similar in many respects, and the relationship between them has been a subject of several phylogenetic papers^2^,^22^,^30^,^31^,^34^ with the definite conclusion that Steninae is a sister group of Euaesthetinae. In our analysis, monophyly of Euaesthetinae and Steninae, as well as their sister relationships were recovered with strong support (PP = 1.00) too.

On the contrary, there is no agreement about a group sister to the Euaesthetinae + Steninae clade. Hansen’s^2^ hypothesis considering the monogeneric subfamily Megalopsidiinae as a sister lineage of Euaesthetinae + Steninae, is rejected by our results. Here, consistent with Grebennikov & Newton^30^, Megalopsidiinae are placed far outside of the Euaesthetinae + Steninae clade, as a rather isolated lineage at the root of Staphylinine-group. Moreover, with all analytical approaches the fossil *Megalopinus* species is resolved as a stem lineage to the recent species of the genus, a possibility already considered in the description of that species^33^. However a wider sampling of recent *Megalopinus* is needed for a sound decision about possible separate generic status of *Megalopinus extinctus*. A morphology and molecular-based study of the subject^30^ placed Scydmaeninae as a sister group of the Euaesthetinae + Steninae clade. However, the latest molecular-only based analysis^31^ did not consistently show sister group relationships for the Euaesthetinae + Steninae clade. It was either sister to Scydmaeninae + Solieriinae group, or each subfamily was a part of a large polytomy. Presumably, a very low sampling of genetic markers (two genes) and insufficient taxon sampling biased those respective studies. Nonetheless, our results are congruent with Grebennikov & Newton^30^ hypothesis that suggests Scydmaeninae as a sister group to the Euaesthetinae + Steninae clade. This is not so surprizing given that we used nearly the same character set. However, this is noteworthy since, unlike Grebennikov & Newton^30^, we did not use larvae but added the newly discovered stem lineages instead.

### Early radiation of Steninae and prey-capture apparatus

Various fossils demonstrate that Steninae and its sister group Euaesthetinae, as well as all other putative sister subfamilies to the Euaesthetinae + Steninae clade (Scydmaeninae, Solieriinae, Megalopsidiinae) coexisted and were well established already in the Late Cretaceous. Moreover, all of them are known from the Burmese amber, which contains a mixture of extinct insect families and the earliest known representatives of modern families, including many seemingly ‘transitional forms’ between ancient and present faunas^47^. All these indicate that diversification into major lineages of various beetle families took place earlier than in the Cretaceous. This is consistent with the hypothesis that all subfamilies of the Staphylinine-group may have originated by the Late Jurassic and were well established in the mid-Cretaceous as shown by several studiese.g. refs 3,30,50,55,56.

Based on alternative phylogenies there are two main hypotheses regarding the development of prey-capture apparatus in Steninae. The first assumes that a slightly protrudable labium of the genus *Dianous* represents a plesiomorphic state, while a more sophisticated adhesive prey-capture apparatus with paraglossae modified into sticky pads in *Stenus* is an apomorphic state. The second hypothesis interprets the lack of such apparatus and sticky pads in *Dianous* as a secondary reduction^24^. *Stenus*-like mouthparts modification of the undescribed Australian genus sister to the genus *Stenus* is apparently synapomorphic to both, while the habitus resemblance of the former to some Euaesthetinae is interpreted as a convergence resulting from their shared forest leaf litter habitat.

Among multiple specimens of the new extinct genus, there are none with a protruded labium (or with open mandibles), either because labium was only slightly elongated in that genus, or because of the preservational bias, since beetles apparently close mandibles and contract labium when trapped by resin. Indeed, there is a whole range of different morphological and anatomical modifications within recent representatives of *Stenus*, some of which even have a vestigial to completely reduced labium, in the most extreme cases^24^,^57^. However, morphological structures that are always associated with the adhesive prey-capture apparatus in *Stenus*, i.e. sticky pads and carina on the mentum, are clearly visible at least in some specimens of our new genus in which mouthparts are well preserved for observation. Discovery of the elaborate adhesive prey-capture apparatus in the stem lineage that presumably is older than the *Stenus-Dianous* crown lineage and that looked like prey-capture apparatus in the recent *Stenus*, is remarkable. It supports the latter hypothesis that *Dianous* is at least partly nested within *Stenus*, and its reduced labium and absence of the sticky pads is a secondary loss. It also suggests that an incredible evolutionary radiation of *Stenus* maybe driven by some factors other than their possession of an effective prey-capture apparatus, since the latter appeared earlier in the evolution of Steninae and did not necessarily cause notable species radiations then. Also we cannot exclude an alternative possible scenario that such prey-capture apparatus evolved more than once in Steninae, with one case documented by the newly discovered stem genus, and another by the recent *Stenus*-lineage.

### Methodological issues

Development of methods for phylogenetic analysis of the morphological data is going much slower than for the genomic data. The most popular analytical approach in morphology is still the maximum parsimony method with the relatively simple criterion for building a phylogenetic tree. Even though likelihood-based methods for the morphological data have been available, they are still rather rarely used, especially for morphological datasets only. Recently it was shown that model-based methods (ML and BI) outperform parsimony despite the fact that the Mk model for discrete morphological data is still very simplistic (e.g. refs 58,59). In the latter studies they show that the most poorly performing method is implied-weight implementations of parsimony. Our contradictive resolution for the Steninae node when applying different analyses shows a necessity of further theoretical and empirical investigation of the performance of different methodological approaches, which is, however, beyond the scope of our paper.

### Future directions

To rigorously test the above-mentioned hypotheses, a well-resolved molecular-based phylogeny of Steninae is needed. Such phylogeny does not exist since the few previous attempts used too few gene markers for the phylogenetic analysis, and were very limited in taxon sampling. Using several diverse gene markers (at least those six already well-proven for beetles including Staphylinidae) sequenced for a good biogeographic representation of different *Stenus* species groups, broader *Dianous* sampling, and inclusion of the undescribed Australian genus should allow to resolve the backbone phylogeny of Steninae. Moreover, combining molecular and morphological data would be particularly important, since it would allow mapping characters associated with the adhesive prey-capture apparatus on a phylogeny to infer their evolution. A targeted study of collections with good holdings of Burmese amber and other fossils will undoubtedly reveal more fossil Steninae whose diversity and morphology, especially of their possible prey-capture organs, will be critical for further investigation of the problem.

## Material and Methods

### Microscopy and illustrations

Both recent and fossil specimens were examined using Leica M205 C and Leica M125 stereoscopes. Drawings were made either freehand or from photographs and digitally inked in Adobe Illustrator C6. Photographs of amber pieces were taken using the Visionary Digital Imaging Systems with a Canon EOS 7D camera. All other photographs were taken with a Canon EOS 6D camera attached to the Leica M205 C stereoscope with the help of EOS Utility 3.4.30.0 software. Photomontage was accomplished using Zerene Stacker (Zerene Systems LLC, 2012) and photos were edited in Adobe Photoshop C6. For the fossil specimens a microCT-scanning was tried using an Xradia MicroXCT x-ray microtomography system (University of Vienna, Department of Theoretical Biology). The software Amira 5.4.3 was used for 3D-visualization and analysis of the data. Unfortunately, the amber structure and composition did not allow extracting more morphological data by microCT-scanning that could be used in our studies. Results of our attempt are shown in the Supplementary Fig. S3.

### Examination and deposition of extant taxa

Ten species in total were prepared for the study; seven of them were included in the analysis. Recent specimens were boiled in 10% KOH to bleach them, which allowed for better observation of the exoskeleton structures. They were then rinsed in distilled water, disarticulated when necessary, and stored/examined in small Petri dishes with glycerine. All specimens scored de novo are kept at the Zoological Museum in Copenhagen, Denmark (ZMUC).

### Examination and deposition of fossil taxa

**Figure 8:**
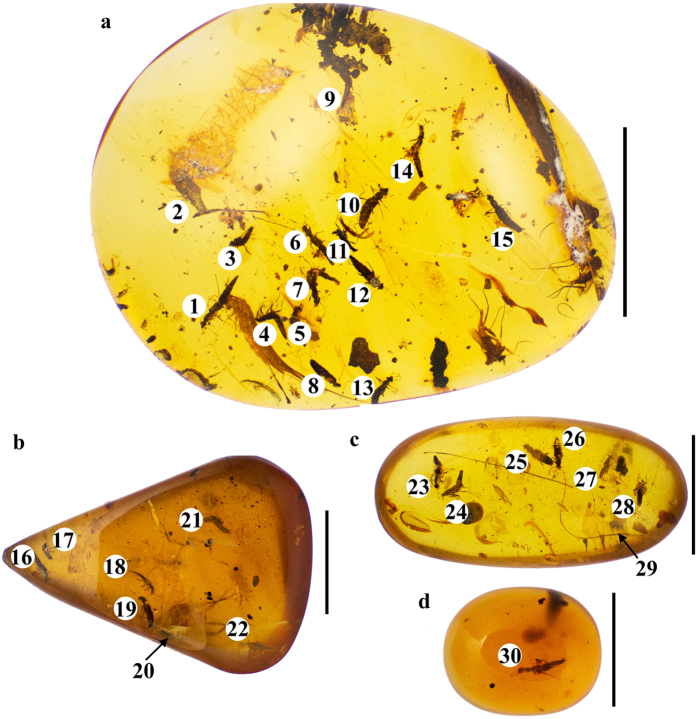
Pieces of Burmese amber with inclusions of Steninae. (**a**) SMNS Bu-119 with 15 specimens. (**b**) AMNH Bu-SY10 with 7 specimens. (**c**) AMNH Bu-SY11 with 7 specimens. (**d**) AMNH Bu-SY12 with 1 specimen. Scale bars = 1.0 cm.

**Figure 9:**
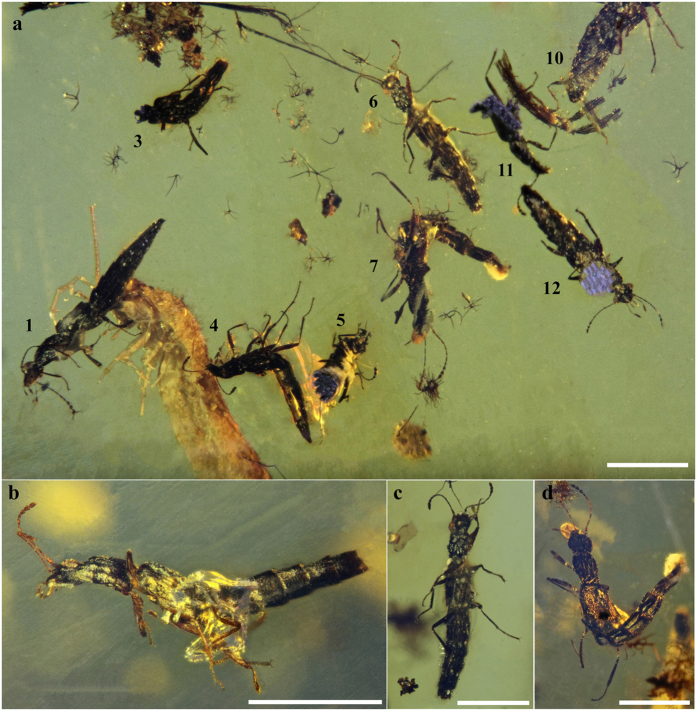
Different way of preservation, piece no SMNS BU-119. (**a**) Several specimens visible from different positions. (**b**) SMNS Bu-119/14, habitus, ventral view, slightly squished, with a bubble covering part of the ventral side. (**c**) SMNS Bu-119/6, habitus, ventral view, highly squished. (**d**) SMNS Bu-119/7, difficultly placed in the amber, visible secretions from the mouth parts and the tip of the abdomen. Scale bars = 1.0 mm.

Our studied material consisted of four pieces of Burmese amber, containing a total of 30 specimens (Fig. 8). Each specimen here was designated with a unique number (e.g. SMNS Bu-119/1) composed from the abbreviation of an institution or a collection that a respective amber piece belongs to (e.g. SMNS), an abbreviation indicating that it is Burmese amber (Bu), an inventory number of a respective amber piece (e.g. 119), and a serial number given here to each specimen (e.g. 1). Of these four amber pieces one is housed in the collection of the State Museum of Natural History at Stuttgart, Germany (SMNS Bu-119). It contains 15 specimens (numbered from 1 to 15, Fig. 2a). The other three pieces, which are originally from the private collection of SY, will be deposited in the American Museum of Natural History, New York, USA. They are registered as AMNH Bu-SY10, AMNH Bu-SY11, and AMNH Bu-SY12 with 7, 7 and 1 specimen, respectively. Those specimens are numbered from 16 to 22 (AMNH Bu-SY10, Fig. 2b), 23 to 29 (AMNH Bu-SY11, Fig. 2c) and 30 (AMNH Bu-SY12, Fig. 2d), respectively. The degree of preservation ranges from a very poorly preserved specimen (Fig. 9a) through slightly distorted (Fig. 9b) or highly deformed (Fig. 9c) to relatively well preserved ones (Fig. 9d) allowing for detailed examination. None of the amber pieces was cut due to complex position of specimens placed in various dimensions, often close to each other in one piece. It would be difficult to cut such ‘dense’ syninclusions without damaging some specimens. At the same time, multiple conspecific inclusions increased chances to observe certain structures in specimens where those were best preserved. This work is registered in ZooBank under LSID (Life Science Identifier) urn:lsid:zoobank.org:pub:B8511C83-29BB-4EEB-B40E-63BBA40C18F7. The ZooBank LSID for the new genus and species are as follows: Festenus LSID, urn:lsid:zoobank.org:act:EF495C63-E977-4FB4-88B8-DD3C177AB446; Festenus robustus LSID, urn:lsid:zoobank.org:act:FF5FAD2E-CAE4-4D26-9532-4BA2CD7BDC85 and Festenus gracilis LSID, urn:lsid:zoobank.org:act:0A167072-A6BD-45F7-BC6F-0790A3471785.

### Taxon sampling and outgroup

The present taxon sample builds on the Clarke & Grebennikov^22^ data matrix as the most adequate for our purpose. Fifteen taxa of their matrix were chosen for our analysis, as follows: *Siagonium* sp. (Piestinae), *Oxyporus* sp. (Oxyporinae), *Nanobius* sp., and *Pseudopsis* sp. (Pseudopsinae), *Megalopinus* sp. (Megalopsidiinae), *Euaesthetus* sp., *Octavius* sp. (*Octavius*PAN in Clarke & Grebennikov^22^), *Stenaesthetus* sp., *Alzadaesthetus* sp. (*A. furcillatus* in Clarke & Grebennikov^22^), *Austroesthetus* sp., *Agnosthaetus* sp., *Mesoaesthetus* sp., *Nothoesthetus* sp. (Euaesthetinae) and two undescribed species of Australian Steninae Gen_AUS_Sp.1 and Gen_AUS_Sp.2 (SteNovAUS1W and SteNovAUS2F, respectively, in Clarke & Grebennikov^22^). To avoid a strong bias in our analysis towards one of the subfamilies, we significantly pruned Euaesthetinae terminals leaving 8 from 21 used in Clarke & Grebennikov^22^. Such alternation required adjustments for some of their characters, such as: removal of characters that became single-state, and addition or removal of character states for some characters. In the Clarke & Grebennikov^22^ data matrix, subfamilies Oxyporinae, Pseudopsinae and Megalopsidiinae were included as a potential sister group to the Euaesthetinae + Steninae clade. Since the next study by Grebennikov & Newton^30^ revealed Scydmaeninae as a sister group to Euaesthetinae + Steninae, and did not resolve sister-group relationships for the presumably related subfamily Solieriinae, we decided to include in our analysis two Scydmaeninae genera: *Euconnus* sp., and *Scydmaenus* sp., and a species of *Solierius*. They were scored from the morphological data matrix developed by Grebennikov & Newton^30^, where the list of characters significantly overlaps with that in Clarke & Grebennikov^22^ used here. For those characters that were not included in the Grebennikov & Newton^30^ character list, additional papers and specimens from the ZMUC collection were used to determine respective character states for the listed Scydmaeninae and Solieriinae taxa. Finally, for tuning the taxon sample towards the goals of our analysis, the following seven recent taxa, not included in either Clarke & Grebennikov^22^ or Grebennikov & Newton^30^ have been added: *Megalopinus punctatus* (Erichson), 1840 (Megalopsidiinae), *Stenus biguttatus* L., *S. cicindeloides* Schaller, *S. nitidiusculus* Stephens, *S. impressus* Germar, *S. bimaculatus* Latreille, and *Dianous coerulescens* Gyllenhal (Steninae). Their characters were scored by examination of the actual specimens. Moreover, the only extinct representative of the subfamily Megalopsidiinae, *Megalopinus extinctus* (specimen number AMNH Bu-SY2) was also scored from the actual holotype specimen. In addition to the fossil specimens of Steninae specifically examined here, we added two fossil species that were scored from the papers where they were originally described: *Prosolierius* Thayer, Newton & Chatzimanolis, 2012 (Solieriinae), and *Octavius electrospinosus* Clarke & Chatzimanolis, 2009 (Euaesthetinae). We did not include an extinct genus *Eocenostenus* into the final analysis because of its poor preservation prohibiting from scoring the majority of characters. Overall, four fossil taxa were added to the matrix. The genus *Siagonium* Kirby & Spence from the subfamily Piestinae that is outside the Staphylinine-group of subfamilies, was chosen as an outgroup because it is phylogenetically the most remote from taxa targeted in our analysis. We also conducted alternative analyses with the genus *Oxyporus* Fabricius (Oxyporinae) as a closer related outgroup, but still phylogenetically far from the Euaesthetinae + Steninae clade based on the previous studies. In order to check the accuracy of our dataset, we also conducted a separate analysis without fossil taxa.

### Phylogenetic analysis

The data matrix that included 134 characters (numbered 1–134) scored for 29 taxa was constructed with Mesquite 3.10^60^. Unknown character states were coded with “?”, inapplicable states with “–”. The character matrix is provided in the Supplementary Text 1.

The maximum parsimony analyses were conducted in TNT 1.5^61^ using the “traditional search” option to find the most parsimonious trees (MPTs) under the following parameters: memory set to hold 100000 trees; tree bisection–reconnection (TBR) branch-swapping algorithm with 1000 replications saving 10 trees per replicate; zero-length branches collapsed after the search. All character states were treated as unordered and equally weighted. Autapomorphic characters were deactivated in the parsimony analysis. We also performed separate analyses under implied weights of characters^62^. Values of the constant of concavity *k* ranged from 1 to 10 representing a gradient from strong to weak down-weighting of the homoplasious characters, respectively. Bremer support was calculated using the TNT Bremer function, using suboptimal trees up to 20 steps longer. Character mapping was made in WinClada v1.00.08^63^ using unambiguous optimization, while trees were annotated in Adobe Illustrator C6.

Bayesian inference was conducted in MrBayes v3.2.6^64^ running on the CIPRES Science Gateway v3.3. (phylo.org), using the Mkv model and default settings for priors. All analyses used four chains (1 cold and 3 heated) and two runs of 15 million generations, each analysis was repeated twice. Autapomorphic characters were included in the data set, and the analyses were conducted with and without gamma distribution. Convergence of both runs was visualized in Tracer v1.6.0^65^, as well as by the examination of PSRF values and average standard deviation of split frequencies in the MrBayes output. Nodes with posterior probability (PP) > 0.95 were considered strongly supported; with PP = 0.90–0.94 moderately supported, and with PP = 0.85–0.89 weakly supported. Nodes with less than 0.85 PP were considered to be unsupported.

Character list is provided in the Supplementary Text 2.

## Additional Information

**How to cite this article**: Żyła, D. *et al*. Cretaceous origin of the unique prey-capture apparatus in mega-diverse genus: stem lineage of Steninae rove beetles discovered in Burmese amber. *Sci. Rep.* **7**, 45904; doi: 10.1038/srep45904 (2017).

**Publisher's note:** Springer Nature remains neutral with regard to jurisdictional claims in published maps and institutional affiliations.

## Supplementary Material

Supplementary Figures and Text

## Figures and Tables

**Figure 5:**
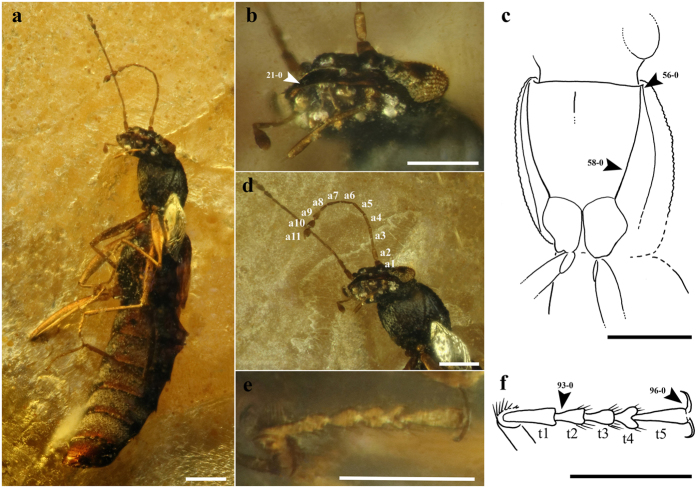
Habitus and body parts of *Festenus robustus* sp. nov., holotype, AMNH Bu-SY10/19. (**a**) Ventral side. (**b**) Ventral view of head. (**c**) Drawing of the ventral side of pronotum. (**d**) Ventral view of pronotum. (**e**) Mesotarsus. (**f**) Drawing of mesotarsus. Abbreviations: a – antennomere, t – tarsomere. Scale bars = 0.2 mm.

**Figure 6:**
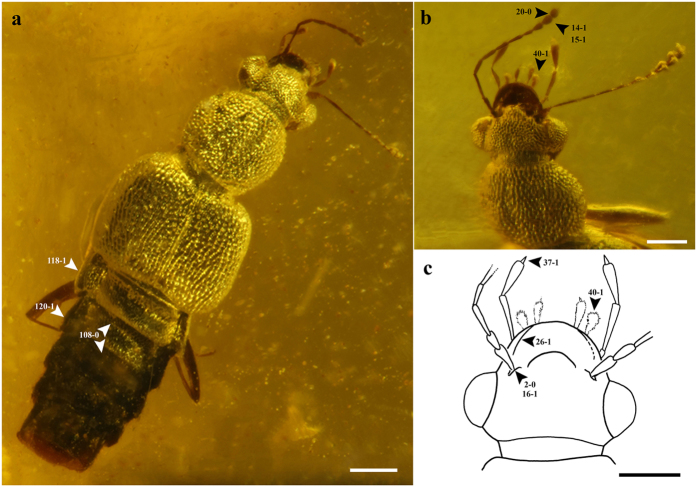
Habitus and body parts of *Festenus robustus* sp. nov., paratype, AMNH Bu-SY11/25. (**a**) Dorsal side. (**b**) Dorsal view of head and pronotum. (**c**) Drawing of head with sticky pads (40-1). Scale bars = 0.2 mm.

**Figure 7:**
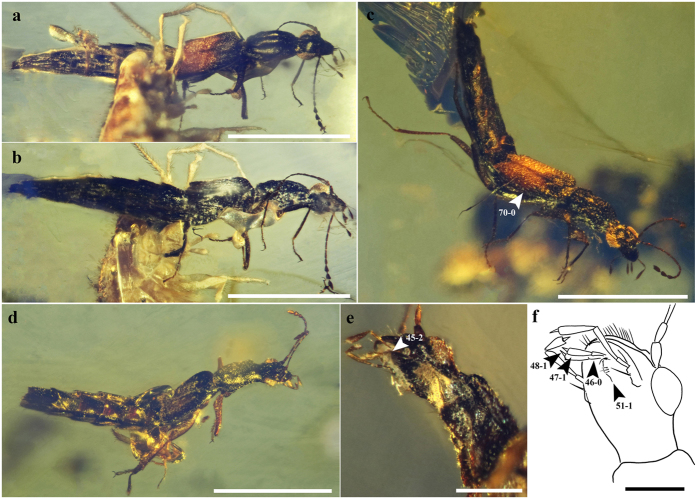
Habitus and body parts of *Festenus gracilis* sp. nov., SMNS Bu-119. (**a**) Holotype, SMNS Bu-119/1, dorsal view. (**b**) Holotype, SMNS Bu-119/1, ventral view. (**c**) Paratype, SMNS Bu-119/9, dorsal view. (**d**) Paratype, SMNS Bu-119/14, dorsal view. (**e**) Paratype, SMNS Bu-119/14, ventral view of head and pronotum. (**f**) Drawing of ventral part of head of specimen SMNS Bu-119/14. Scale bars = 1.0 mm for (**a**–**d**), 0.2 mm for (**e**,**f**).
