# Supplementary material for: Cretaceous origin of the unique prey-capture apparatus in mega-diverse genus: stem lineage of Steninae rove beetles discovered in Burmese amber
Source: Sci Rep. 2017 Apr 11;7:45904. doi: 10.1038/srep45904 (PMC5387741; doi:10.1038/srep45904)
Supplement: Supplementary Figures and Text [file srep45904-s1.pdf]

Cretaceous origin of the unique prey-capture apparatus in mega-diverse genus: stem lineage of Steninae rove beetles discovered in Burmese amber

Dagmara Żyła, Shûhei Yamamoto, Karin Wolf-Schwenninger, Alexey Solodovnikov

Supplementary figures

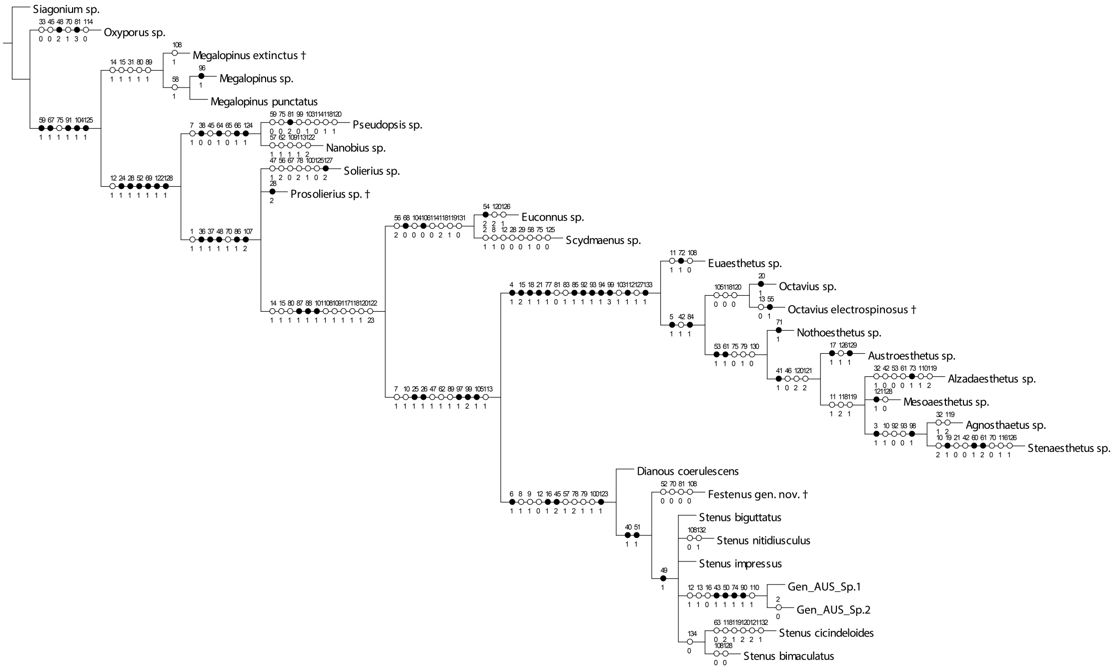

Figure S1. Most parsimonious tree under implied weights (IW) and a *k*-value = 3. Circles with numbers along branches indicate unambiguously optimized synapomorphies (autapomorphies for terminal branches): black, unique changes; white, homoplasious changes; character numbers above circles, character state numbers below circles.

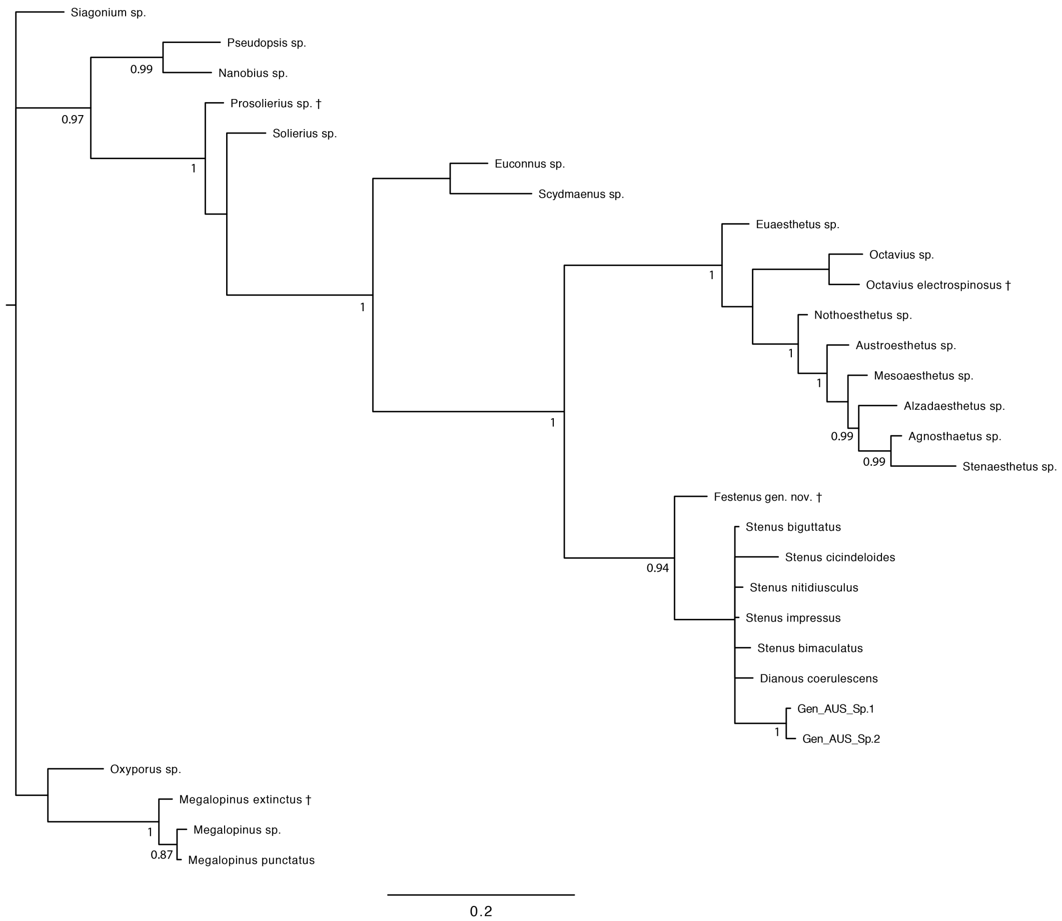

Figure S2. Fifty percent consensus tree from a Bayesian analysis with equal rates. Posterior probabilities greater than 0.84 reported below the corresponding node.

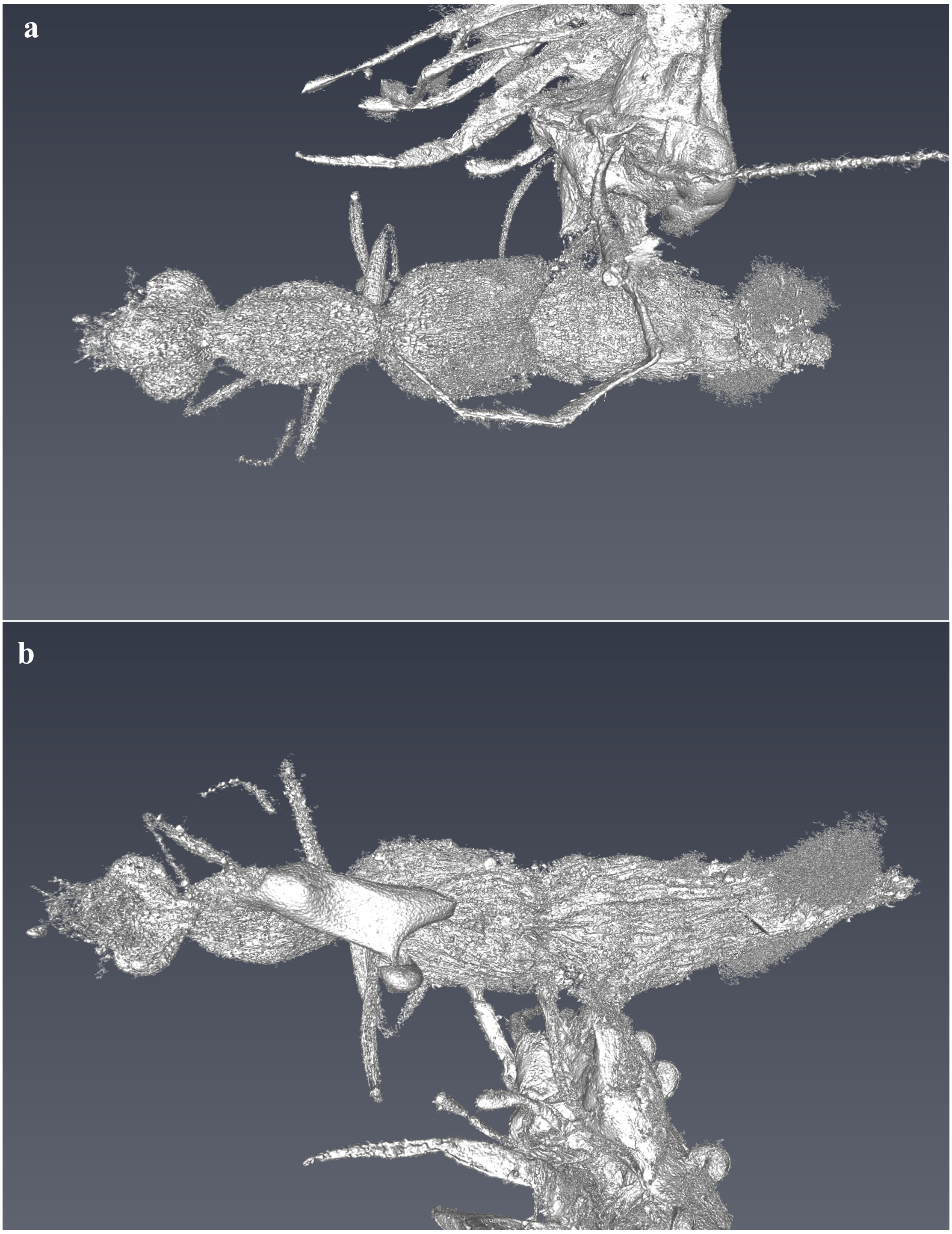

Figure S3. Micro-CT scans of *Festenus gracilis* sp. nov., holotype, SMNS BU-119/1. a. Dorsal view. b. Ventral view.

Supplementary text

1. Data matrix

Siagonium_sp.
0100000100200000000000000000000001000010001001000000000000000000001000010000000000000000
000000000000000000000001100000010010000000000000001000
Oxyporus_sp.
0000000000100000000000000000000000000001000000000200000000000000001001010000000000310
000000000000000010000000100000000000000000000000000000
Megalopinus_sp.
000000000110001100000000000000110100001000000100000000100011000101011000000?00001011
0000010100001001000010100000011000000000001000000101
Megalopinus_punctatus
000000000110001100000000000000110100001000000?1000000?01000110001010110000000100001011
0000010100000001000010100000011000000000001000000101
Megalopinus_extinctus
000000?????001100000??00000??1???001?0000?1000000???0000100010??110000?0100001?????0
0?10100000001000010101?00??100?00000???????????
Pseudopsis_sp.
0000000100201100000000010001000010000000000000000000001000000000001011110000000000000200
0000000100000000000011010000000000001010101 10?101100?
Nanobius_sp.
0000000100201100000000010001100010000000000000000000001000010100111011110000010000000000
00000001000000010000101001000110000000201101 1011000
Solierius_sp.
010000000?201?0000?00??10001100?1??111?0000?1?110001010200100000?0011100001??2001?0?
0100001000000?1100?1012000000100000001000021 0011?1
Prosolierius_sp.
01000???????1?0000?00??100?2???????11??0?????101??????00?010???0???1110000???????????????
01000000????0?10120000??????0000??0?00?0????1
Euconnus_sp.
010000000?201?1100?00??10001111?0??111?0000?1?0100010202001 0?000?0101100001??0?11?0?0
111001000000?1011?000-1100000101212030011010101?1
Scydmaenus_sp.
01100-
001?200?1100?00??10000000?1??111?0000?1?01000101020110?000?0101100000??0?11?0?0111001
000000?1011?000-110000000121102000001 0001?1
Stenus_biguttatus
0110001111100011100000001111111100101111100012011101101011110011010111100000000211100
0011110100010102111011121100011101101031010010 11001
Stenus_cicindeloides
0110001111100011100000001111111100101111100012011101101011110010010111100000000211100
0011110100010102 1?101112110001110121223101001011100
Stenus_nitidiusculus
0110001111100011100000001111111100101111100012011101101011110011010111100000000211100
0011110100010102111011120100011101101031010010 11101
Stenus_impressus
0110001111100011100000001111111100101111100012011101101011110011010111100000000211100
0011110100010102111011121100011101101031010010 11001
Stenus_bimaculatus
0110001111100011100000001111111100101111100012011101101011110011010111100000000211100
0011110100010102111011120100011101101031010000 11000
Dianous_coerulescens
0110001111100011100000001111111100101111000002011000101011110011010111100000000211100
0011110100010102111011121100011101101031010010 11001
'Gen_AUS_Sp.1'
0110001111101111000000001111111100101111100112011111101011110011010111100010000211100
0011111100010102111011121110011101101031010010 11001

'Gen_AUS_Sp.2'
01000011111011110000000111111110010111110011201111110101111001101011110001000211100
00111111000101021110111211100111011010310100101100 1
Festenus_gen._nov.
010000??????001110?00??111????1???0111?1?00?201100100?00?0100?101?111000?01?02110????1
1?101000101?21?1011120100??1?0?10103????0???????
Alzadaesthetus_sp.
11001101000111120010000111011111010111101000101100010100001001101011110010001111001
1111110111110103010111121110111101222230010110 01011
Austroesthetus_sp.
11001101000011120110000111011110010111101100101100011100001011101011110000001111001
111111011111010301011112110011110110223001111101011
Mesoaesthetus_sp.
11001101000111120010000111011110010111101100101100011100001011101011110000001111001
111111011111010301011112110011110121213001010001011
Nothoesthetus_sp.
11001101000011120010000111011110010111100100111100011100001011101011111000001111001
111111011111010301011112110011110110103001011001011
Euaesthetus_sp.
11001001000111120010000111111110010111100000111100010100001001101011110100111101001
011111011111010301011112010011110110103001011011111
Octavius_sp.
11001101102011120010111111011110010111100100111100010100001001101011110000111101001
211101011111010301011012110111111100001001111010011
Octavius_electrospinosus
11001???????10120??00??111????????011???????1111??????1???????????1111????1?????0?????1?1
01111?01?3???1?012??????1???0000???????????11
Agnosthaetus_sp.
11011101001111120010000111011111010111101100101100011100001011101011110000001111001
111111010011011301011112110011110122223001011001011
Stenaesthetus_sp.
01011101002111120011000111011110010111101000101100011100001121101011100000001111001
111111010011011301011112110011111121223001111001011

2. List of characters

Most character systems below are illustrated in the following papers: Orousset (Supplementary reference 1), (structure of mesothoracic apodemes); Clarke & Grebennikov (22) (abbreviated here as C&G); Grebennikov & Newton (30); Weide et al. (Supplementary reference 2) (tentorium structures) and Herman (Supplementary reference 3) (medial lobes of labium). Autapomorphic characters are marked with asterisk *.

Head capsule.

1. Epistomal (frontoclypeal) suture: (0) present; (1) absent.
2. Location of antennal insertion, in dorsal view: (0) near frontal margin of head, anterior to eye (Fig. 6c; C&G: Fig. 11A–D); (1) on frons between eyes (C&G: Fig. 11E).
3. Dorsolateral carina of head: (0) absent (C&G: Fig. 11C–E); (1) present at least anteriorly (C&G: Fig. 11A, arrow, B, bottom arrow).
4. Postoccipital nuchal region: (0) absent; (1) present (Orousset: Fig. 4).
5. Gular sutures: (0) separate; (1) united along most of length; (2) united anteriorly only.
6. Apodemes arising from interantennal pits: (0) absent; (1) present.
7. Dorsal tentorial arms (DTA in Weide et al.: Figs 2, 3, 12): (0) not fused with cranium; (1) fused with cranium.
8. Tentorial bridge (TB in Weide et al.: Figs 2–6, 12): (0) present; (1) absent.
9. Tentorial loop: (0) absent; (1) present.
10. Corporotentorium/laminatentorium (LT in Weide et al.: Figs 2–6, 12): (0) split; (1) absent; (2) fused.
11. Dorsomedian nuchal phragma: (0) absent; (1) present.
12. Ommatidia structure: (0) facets hexagonal and flat; eye surface smooth; (1) facets round and strongly convex; eye surface botryoidal (C&G: Fig. 11F).
13. Long interfacetal ocular setae: (0) absent; (1) present (C&G: Fig. 11F).
14. Antennal club: (0) absent; (1) present (Fig. 5b; C&G: Fig. 11G).
15. Number of antennomeres in antennal club: (0) zero, club absent; (1) three (Fig. 6b); (2) two.
16. Condyle of first antennomere in dorsal view: (0) concealed (C&G: Fig. 11A, C–E); (1) exposed (Fig. 6c; C&G: Fig. 11B, top arrow).

17.* Apex of antennomere 10: (0) not concave to receive antennomere 11; (1) concave to receive antennomere 11.

18. Differentiated setae on antennomere 10: (0) absent; (1) present (C&G: Fig. 11G, H, arrows).

19.* Length of antennomeres 9–11: (0) each less than 3x maximum width (C&G: Fig. 11G); (1) each greater than 3x maximum width.

20.* Antennomeres 10 and 11: (0) separated by antennal stem (Fig. 6b); (1) partly fused, antennal stem absent (C&G: Fig. 11G).

21 Anterior margin of labrum: (0) smooth (Fig. 6b; C&G: Fig. 11A); (1) denticulate or serrate (C&G: Fig. 11C, I, M).

22.* Surface of epipharynx: (0) flat, without furrows; (1) distinctly longitudinally furrowed (C&G: Fig. 11I, J, left arrow).

23.* Epipharyngeal marginal setae: (0) absent; (1) present (C&G: Fig. 11J, right arrow).

24. Frontoclypeal–labral junction: (0) not visible in dorsal view, labral attachment concealed beneath frontoclypeal margin; (1) visible in dorsal view, labrum attached to frontoclypeal margin (C&G: Fig. 11A, C, E).

25. Mandibular structure: (0) robust; (1) slender, falciform (C&G: Fig. 11L).

26. Mandibles when closed: (0) tips fully exposed; (1) tips concealed beneath labrum (Fig. 6c; C&G: Fig. 11K).

27. Inner edge of mandibles posterior to preapical tooth: (0) smooth; (1) serrated (C&G: Fig. 11L).

28. Preapical mandibular teeth: (0) asymmetrical in number; (1) symmetrical in number (C&G: Fig. 11L); (2) absent.

29. Maximum number of preapical teeth on inner margin of mandibles: (0) two; (1) one.

30. Mandibular prostheca: (0) present; (1) absent.

31. Mandibular molar lobe: (0) present; (1) absent.

32. Spine on lateral edge of galea: (0) absent (C&G: Fig. 12B); (1) present (C&G: Fig. 11M, left arrow).

33. Apical unarticulated spine of lacinia: (0) absent; (1) present.

34. Cluster of digitiform sensilla on outer side of maxillary palpomere 3: (0) absent; (1) present (C&G: Fig. 11N, arrow).

35.* Pair of papillate sensilla at apex of maxillary palpomere 3: (0) absent; (1) present (C&G: Fig. 11O, arrow).

36. Setation of maxillary palpomere 3: (0) glabrous except for few scattered macrosetae; (1) very densely setose and without macrosetae (C&G: Fig. 11N).

37. Maxillary palpomere 4: (0) well developed, fully sclerotized; (1) minute, hyaline (Fig. 5c; C&G: Fig. 11N, O).

38. Medial lobes of labium: (0) present (Herman: Fig. 8); (1) absent.

39. Two pairs of setae on anterior margin of labium: (0) absent; (1) present (C&G: Figs 11I, M, right arrow; 12A, right arrow, B).

40. Adhesive cushions of labium: (0) absent; (1) present (Fig. 6b, C; C&G: Figs 11L, right arrow; 12A, left arrow).

41. Digitiform processes of labium: (0) absent; (1) present (C&G: Fig. 12B, top arrow; D, left arrow).

42. Mesal notch in apex of labium: (0) absent; (1) present (C&G: Fig. 11I, M).

43. Mesal mound at apex of labium: (0) absent; (1) present (C&G: Fig. 12A, right arrow).

44. (C&G: 45) Lateral rows or combs of setae on hypopharynx: (0) present (C&G: Fig. 12D, right arrow); (1) absent.

45. (C&G: 46) Insertion location and proximity of labial palps: (0) more or less contiguous and nearer to base than to apex of labium; (1) widely separated at sides of labium (C&G: Figs 11I, M; 12B, C); (2) almost contiguous and nearer to medial apex than to sides of labium (Fig. 7e; C&G: Fig. 12A).

46. (C&G: 47) Labial palpomere 1: (0) elongate, half as long as, to slightly longer than, palpomere 2 (Fig. 7f; C&G: Figs 11M; 12A, B); (1) much shorter than half length of palpomere 2 (the latter sessile to subsessile) (C&G: Figs 11I; 12C).

47. (C&G: 48) Labial palpomere 2: (0) unmodified, similar in shape to palpomere 1; (1) strongly expanded, subglobular or subfusiform (Fig. 7f; C&G: Figs 11I, M; 12A-C).

48. (C&G: 49) Labial palpomere 3: (0) well developed, fully sclerotized; (1) acicular, hyaline (Fig. 7f; C&G: Figs 11I, M; 12A, C); (2) moderately to strongly expanded apically, subtriangular (autapomorphic for Oxyporinae).

49. (C&G: 50) Prementum: (0) normal (C&G: e.g. Figs 11I, M; 12B, C); (1) modified, elongated into eversible rod-like structure (C&G: Fig. 11L).

50. (C&G: 51) Mentum: (0) entire surface in same plane (C&G: Figs 11I; 12B); (1) transversely divided near middle by a ridge, with anterior half deflected vertically (C&G: Fig. 11L, left arrow).
51. (C&G: 52) Lateral palpomere rests on mentum divided by medial longitudinal carina: (0) absent; (1) present (Fig. 7f; C&G: Fig. 11K, arrow).
52. (C&G: 53) Submentum and gula: (0) separated by suture located significantly anterior to posterior tentorial pits; (1) fused.
53. (C&G: 54) Submental transverse carina: (0) absent; (1) present (C&G: Fig. 12B, bottom arrow).

Thorax and legs.

54. (C&G: 55) Cervical sclerite: (0) large; (1) small and very slender.
55.* (C&G: 56) Anterior margin of prosternum: (0) smooth; (1) deeply notched (C&G: Fig. 12E, top arrow).
56. (C&G: 57) Pronotal marginal carina: (0) not meeting pronotosternal suture, reaching anterolateral prothoracic margin (Fig. 5c; C&G: Fig. 12F, arrow); (1) meeting pronotosternal suture anterolaterally (C&G: Fig. 12G, left arrow), not reaching anterior prothoracic margin.
57. (C&G: 58) Ventral hypomeral marginal carina: (0) present (C&G: Fig. 12E, bottom arrow); (1) absent.
58. (C&G: 59) Pronotosternal suture: (0) present and complete (Fig. 5c; C&G: Fig. 12E, middle arrow); (1) absent or very incomplete and evident only posteriorly near coxal cavity (C&G: Fig. 12H).
59. (C&G: 60) Protrochantin: (0) exposed; (1) concealed.
60.* (C&G: 61) Prosternal callosity (usually depigmented): (0) absent; (1) present (C&G: Fig. 12I, top arrow).
61. (C&G: 62) Anteprocoxal carina: (0) absent (C&G: Fig. 12E, H, K); (1) present, transversely arcuate (C&G: Fig. 12J, left arrow); (2) present and divided, with each side directed anteroobliquely (C&G: Fig. 12I, bottom arrow).
62. (C&G: 63) Anteprocoxal lobes: (0) absent (C&G: Fig. 12K); (1) present (C&G: Fig. 12J, right arrow).
63. (C&G: 64) Procoxal mesial surface: (0) without carina-delimited groove; (1) with carina-delimited groove (C&G: Fig. 12K, arrow).

64. (C&G: 65) Longitudinal carinae or costae on pronotum and elytra: (0) absent; (1) present (Thayer (4) lists state 1 as an apomorphy for Pseudopsinae).
65. (C&G: 66) Mesothoracic spiracles in ventral view: (0) exposed; (1) concealed by pronotum.
66. (C&G: 67) Antemesoventral sclerite or sclerites: (0) absent; (1) present (one or two sclerites are positioned ventrally in the membrane between pro- and mesothorax, and are separated from the mesothoracic spiracles).
67. (C&G: 68) Scutellum: (0) mostly visible in dorsal view; (1) mostly to entirely concealed by the posterior pronotal edge (C&G: Fig. 12L).
68. (G&N: 141) Elytral length relative to abdomen, viewed from above: (0) slightly truncate (1-2 terga exposed); (1) short (6-7 terga exposed).
69. Elytral striae: (0) present; (1) absent.
70. Elytral epipleural keel: (0) present (Fig. 7c; C&G: Fig. 12M, left arrow); (1) absent.
71.* Elytral epipleural fold: (0) absent; (1) present (C&G: Fig. 13A, arrow).
72.* Basal spine of elytral marginal ridge: (0) absent; (1) present (C&G: Fig. 13B, arrow).
73.* Underside of elytra: (0) smooth; (1) densely tuberculate (C&G: Fig. 13C).
74. Procoxal rests of mesoventrite: (0) absent; (1) present (C&G: Fig. 13E, top arrow).
75. Midlongitudinal carina of mesoventrite: (0) absent; (1) present (C&G: Fig. 13 F, middle arrow).
76. Oblique carina of mesoventrite: (0) absent; (1) present (C&G: Fig. 13F, right arrow).
77. Mesothoracic pleural suture: (0) present (C&G: Fig. 13E, left arrow); (1) absent, (C&G: Fig. 13D, F).
78. Mesothoracic anapleural suture: (0) present at both anterior and posterior ends; (1) present only posteriorly (C&G: Fig. 13D, left arrow, F, bottom left arrow); (2) absent (C&G: Fig. 13E).
79. Transverse carina on side of mesothorax: (0) present (C&G: Fig. 13F, top left arrow); (1) absent.
80. Mesotrochantin: (0) exposed; (1) concealed.
81. Intermesocoxal process of mesoventrite: (0) overlapping intermesocoxal process of metaventrite ventrally (C&G: Fig. 13D, middle arrow, F); (1) with apex abutting

apex of intermesocoxal process of metaventrite (C&G: Fig. 13E, bottom arrow); (2) cariniform, reduced; (3) absent (coxae widely separated by anterior part of metaventrite).

82. Mesothoracic apodemes: (0) projecting anteriorly, free from pleural phragma (Orousset: fig. 299); (1) projecting anterodorsally, partly fused to pleural phragma.
83. Shape of mesothoracic apodemes: (0) elbowed; (1) straight (Orousset: fig. 299).
84. Fusion of mesothoracic apodemes with mesoventrite: (0) completely free from mesoventrite after basal point of attachment to mesoventrite; (1) partly fused anteriorly to mesoventrite; (2) fused tomesoventrite along entire length.
85. Apical muscle disc of mesothoracic apodemes: (0) absent; (1) present (e Orousset: fig. 299).
86. Meso-metaventral suture: (0) present dorsal to mesocoxae (or between mesocoxae, as in *Oxyporus*); (1) absent dorsal to mesocoxae.
87. Mesal posterior lobes of metaventrite: (0) absent; (1) present.
88. Stem of metendosternite: (0) present; (1) absent.
89. Tibial apical spurs: (0) well developed (C&G: Fig. 13G, right arrow); (1) reduced (C&G: Fig. 13H, right arrows).
90. Protibia: (0) normal, rounded; (1) distinctly expanded and concave ventrally (C&G: Fig. 13I, arrow).
91. Protibial external spines: (0) present (C&G: Fig. 13G, top arrow); (1) absent.
92. (C&G: 93) Protarsomeres 1 and 2: (0) articulated (C&G: Fig. 13L, arrow); (1) fused (C&G: Fig. 13H, left arrow).
93. (C&G: 94) Mesotarsomeres 1 and 2: (0) articulated (Fig. 5f); (1) fused.
94. (C&G: 95) Metatarsomeres 1 and 2: (0) articulated; (1) fused.
95. (C&G: 96) Ventral process projecting over empodium: (0) absent (C&G: Fig. 13N, right arrow); (1) present (C&G: Fig. 13M, arrow).
96.* (C&G: 97) Tarsal claws: (0) smooth (Fig. 5f; C&G: Fig. 13G, M); (1) serrate basoventrally (C&G: Fig. 13N, top arrow).
97. (C&G: 98) Empodial setae: (0) present (C&G: Fig. 13G, left arrow, N, middle arrow); (1) absent (C&G: Fig. 13M).
98. (C&G: 99) Mesocoxal mesial surface: (0) without carina-delimited groove; (1) with carina-delimited groove (C&G: Fig. 13D, right arrow).
99. (C&G: 100) Metacoxae: (0) strongly expanded laterally and posteriorly; with posterolateral edge reaching and contiguous with dorsal edge of metepimeron

(C&G: Fig. 13O, arrow); (1) strongly expanded laterally but not posteriorly; with posterior edge contiguous with posterior margin of metepimeron (C&G: Fig. 14A, arrow); (2) not strongly expanded laterally; subtriangular to subconical, length subequal to greatest width; posterior face more or less rounded, not forming distinct edge (C&G: Fig. 14B, arrow); (3) moderately expanded posterolaterally; subtriangular to subconical with posterolateral edge forming sharpened flange laterally (C&G: Fig. 14C, E, upper left arrow), reaching and contiguous with posterior margin of metepimeron (C&G: Fig. 14C, right arrow).

100. (C&G: 101) Posterior face of metacoxae: (0) oblique (C&G: Figs 13O; 14A, C); (1) vertical (C&G: Fig. 14B, arrow, D, arrow).

101. (C&G: 102) Mesal articulations of metacoxae: (0) approximate, close to mesal edges of metacoxae; (1) widely separated and on ventral side of metacoxae.

102. (C&G: 103) Proximity of mesal edges of metacoxae: (0) contiguous or only very narrowly separated anteriorly (C&G: Fig. 14E, right arrow); (1) widely separated anteriorly, usually by a distance of more than half the length of the metacoxae.

103. (C&G: 104) Line of macrosetae on posterolateral edges of metacoxae: (0) absent; (1) present (C&G: Fig. 14C, left arrow, E, bottom arrow).

Abdomen (excluding genital segment and genitalia)

104. (C&G: 105) Wing-folding microtrichia patches on tergite IV: (0) present (C&G: Fig. 14F, middle arrow); (1) absent.

105. (C&G: 106) Attachment of abdominal intersegmental membrane to preceding segment: (0) apical (C&G: Fig. 14G, I); (1) preapical (C&G: Fig. 14H, J).

106. (G&N: 183) Intersegmental membrane sclerites: (0) absent; (1) present.

107. Shape of intersegmental membrane sclerites: (0) quadrangular; (1) irregular; (2) hexagonal.

108. Anterior transverse basal carina of abdominal tergites IV–VII: (0) present (Fig. 6a; C&G: Fig. 14F, top arrow); (1) absent.

109. Basolateral ridges of abdominal tergites: (0) present (C&G: Fig. 14F, left arrow); (1) absent.

110. Deep arcuate carinae at base of abdominal tergites: (0) absent; (1) present (C&G: Fig. 14H, right arrow).

111.* Apicolateral spines of abdominal tergites III–VI: (0) absent; (1) present (C&G: Fig. 14I, arrow).

112. Spiracles of abdominal segment I: (0) placed in membrane beside tergite I; (1) placed in tergite I.

113. Spiracles of abdominal segment II: (0) placed in membrane beside tergite II; (1) placed in tergite II.

114. Intercoxal carina of sternites II/III: (0) absent; (1) present (C&G: Fig. 14J, right arrow).

115. Longitudinal carina at sides of sternite III: (0) absent; (1) present (C&G: Fig. 14J, middle arrow).

116. Paramedial carinae of sternite III: (0) absent; (1) present (C&G: Fig. 14J, bottom left arrow).

117. Parasclerites on each side of segment II: (0) one present (C&G: Fig. 14F, top left arrow); (1) absent.

118. Parasclerites on each side of segment III: (0) two present (C&G: Fig. 14F); (1) one present (Fig. 7A; C&G: Fig. 14H, arrow); (2) absent (C&G: Fig. 14J).

119. Tergum and sternum of segment III: (0) separated by parasclerites (C&G: Fig. 14F, H, I); (1) separated by suture (C&G: Fig. 14J, top arrow); (2) fused into solid ring.

120. Parasclerites on each side of segments IV–VI: (0) two present (C&G: Fig. 14F); (1) one present (Fig. 7A); (2) absent (C&G: Fig. 14J).

121. Tergum and sternum of segments IV–VI: (0) separated by parasclerites (C&G: Fig. 14F); (1) separated by suture; (2) fused into solid ring.

122. Parasclerites on each side of segment VII: (0) two present, longitudinally separated; (1) two present, one more anterior to other (C&G: Fig. 14G, arrows); (2) one present; (3) absent.

123. Paired pygidial defence glands opening into rectum: (0) absent; (1) present.

Genitalia.

124. Stridulatory file of tergite IX: (0) absent; (1) present.

125. Tergite IX in male: (0) divided (C&G: Fig. 14K, arrow); (1) entire (C&G: Fig. 14L).

126. Tergite IX in male: (0) separated from tergite X at least laterally (C&G: Fig. 14K, L); (1) fused to tergite X, or absent (C&G: Fig. 14M).

127. Apex of sternite IX in male: (0) truncate or emarginate, not acutely produced (C&G: Fig. 14N, arrow); (1) acutely produced into medial spine (C&G: Fig. 14O, arrow).

128. Aedeagus when retracted in abdomen: (0) with median foramen left lateral; (1) with median foramen dorsal.

129.* Parameres: (0) normal, unilobed; (1) bilobed.

130. Tergite IX in female: (0) completely divided by tergite X, or connected at most by thread-like cuticular or membranous strip (C&G: Fig. 15A, arrow); (1) not divided by, and forming elongate bridge in front of, tergite X (C&G: Fig. 15B).

131. (C&G: 132) Female intergonopodal sclerite: (0) present (C&G: Fig. 15D, left arrow); (1) absent.

132. (C&G: 133) First and second gonocoxites: (0) distinctly separated by suture (C&G: Fig. 15D, right arrow); (1) fused ipsilaterally (Thayer, 2005) (C&G: Fig. 15E).

133. (C&G: 134) Mesal edge of second gonocoxite: (0) not produced into spine (C&G: Fig. 15E, arrow); (1) produced into spine (C&G: Fig. 15A, right arrow).

134. (C&G: 135) Gonostyle: (0) present (C&G: Fig. 15F, arrow); (1) absent.

Supplementary references

1. Orousset, J. Un nouveau genre d'Euaesthetinae africain: *Macroturellus pulcher* n. gen., n. sp. *Bulletin de la Socie´te´ Entomologique de France* **91**, 219–227 (1987).

2. Weide, D., Thayer, M. K. & Betz, O. Comparative morphology of the tentorium and hypopharyngeal-premental sclerites in sporophagous and non-sporophagous adult Aleocharinae (Coleoptera: Staphylinidae). *Acta Zoologica* **95**, 84–110 (2014).

3. Herman, L. H. Revision and phylogeny of the monogeneric subfamily Pseudopsinae for the world (Staphylinidae, Coleoptera). *Bull. Am. Mus. Nat. Hist.* **155**, 243–317 (1975).
